# Expression of *p53* in human adipose tissue correlates positively with *FAS* and BMI

**DOI:** 10.1038/s41366-024-01691-4

**Published:** 2024-12-01

**Authors:** Stephan Wueest, Chiara Scaffidi, Pim P. van Krieken, Nils K. Konrad, Christian Koch, Ioannis G. Lempesis, Jonas Fullin, Konstantinos N. Manolopoulos, Steffen Böttcher, Gijs H. Goossens, Matthias Blüher, Daniel Konrad

**Affiliations:** 1 Division of Pediatric Endocrinology and Diabetology, University Children’s Hospital, University of Zurich, Zurich, Switzerland; 2 Children’s Research Center, University Children’s Hospital, University of Zurich, Zurich, Switzerland; 3 Department of Medical Oncology and Hematology, University of Zurich and University Hospital Zurich, Zurich, Switzerland; 4 Department of Human Biology, Institute of Nutrition and Translational Research in Metabolism (NUTRIM), Maastricht University Medical Center+, Maastricht, The Netherlands; 5 Institute of Metabolism and Systems Research (IMSR), College of Medical and Dental Sciences, University of Birmingham, Birmingham, UK; 6 Centre for Endocrinology, Diabetes and Metabolism, Birmingham Health Partners, Birmingham, UK; 7 Medical Department III—Endocrinology, Nephrology, Rheumatology, University of Leipzig Medical Center, Leipzig, Germany; 8 Helmholtz Institute for Metabolic, Obesity and Vascular Research (HI-MAG) of the Helmholtz Zentrum München at the University of Leipzig and University Hospital Leipzig, Leipzig, Germany; 9 Zurich Center for Integrative Human Physiology, University of Zurich, Zurich, Switzerland

**Keywords:** Obesity, Fat metabolism

## Abstract

Activation of Fas (CD95) in adipocytes inhibits browning and may contribute to body weight gain in mice. Moreover, Fas expression in white adipose tissue (WAT) correlates positively with body mass index (BMI) in humans. However, molecular pathways involved in the inhibitory effect of Fas on energy metabolism remain incompletely understood. Herein, we report that protein levels of the tumor suppressor p53 were reduced in primary white adipocytes of adipocyte-specific Fas-knockout mice. Moreover, Fas ligand (FasL) treatment increased p53 concentrations in cultured adipocytes and decreased mitochondrial oxygen consumption in control but not in p53-depleted cells, indicating that Fas activation reduces energy expenditure in a p53-dependent manner. In line, in differentiated human mesenchymal stem cells and WAT derived from different anatomical depots, *FAS* expression was positively associated with *p53*. Furthermore, *p53* expression in human subcutaneous and visceral WAT correlated positively with BMI, whereas its expression in visceral WAT was inversely associated with insulin sensitivity (as assessed by hyperinsulinemic-euglycemic clamp). Taken together, our data suggest that Fas regulates p53 expression in adipocytes, and may thereby affect body weight gain and insulin sensitivity.

## Introduction

We recently found that adipocyte-expressed Fas (CD95) may be a therapeutic target to reduce obesity and associated diseases [1]. In particular, high-fat diet (HFD)-fed adipocyte-specific Fas knockout mice displayed reduced inflammation and elevated browning of WAT, increased insulin sensitivity, whole body energy expenditure and reduced body weight gain compared to control littermates. In line, *FAS* expression in human WAT correlated positively with adiposity, indicating that the negative effect of Fas may be conserved between species [1].

Similar to Fas [1–3], expression of the tumor suppressor p53 was increased in WAT of mice and men with obesity, and may contribute to the development of WAT inflammation and, consequently, insulin resistance [4–6]. In addition, p53 may blunt energy expenditure in WAT as suggested by increased oxygen consumption rate (OCR) in p53-depleted white adipocytes [7]. Since Fas activation may increase the abundance of the tumor suppressor p53 [8], we aimed to investigate whether the detrimental effects of Fas on glucose homeostasis and energy metabolism may at least partly be mediated by p53.

## Material and methods

### Humans

Paired abdominal and femoral subcutaneous AT needle biopsies were obtained from 18 postmenopausal women (BMI range: 21.2–40.6 kg/m^2^, age range: 50–62 years) and *p53* mRNA expression was determined as described [9]. The UK Health Research Authority National Health System Research Ethics Committee approved the present study (approval no. 18/NW/0392). For further details please see Supplementary Material and Methods.

In a cross-sectional study, in which 302 individuals participated (205 women, 97 men; BMI range: 16.9–85.5 kg/m², age range: 16–90 years), we investigated *FAS* and *p53* mRNA expression in subcutaneous and/or visceral WAT samples collected during elective laparoscopic abdominal surgery as described previously [10]. Hyperinsulinemic-euglycemic clamps were performed in a subset of individuals as described therein. The study was approved by the Ethics Committee of the University of Leipzig (approval no: 159-12-21052012), and performed in accordance to the declaration of Helsinki. All subjects gave written informed consent before taking part in this study. The probes (Life technologies, Darmstadt, Germany) for p53 (Hs01034249_m1) and *HPRT1* (Hs01003267_m1) span exon-exon boundaries.

### Isolation of white adipocytes

Adipocyte-specific Fas knockout mice were generated and housed as described [1]. All protocols conformed to the Swiss animal protection laws and were approved by the Cantonal Veterinary Office in Zurich, Switzerland. Group size was determined based on previous experiments performed in our laboratory. Group allocation was determined by the genotype. Experimenters were not blinded to group allocations. White adipocytes were isolated from 26-week old HFD-fed male mice as previously described [11].

### Mitochondrial oxygen consumption in adipocytes

Differentiated subcutaneous adipocytes were treated with 0.4 ng/ml FasL or vehicle for 72 h. After 66 h, 1 μmol/l isoproterenol was added to all wells for 5 h to induce browning. Subsequently, medium was replaced to Seahorse XF DMEM Medium pH 7.4 supplemented with 25 mM glucose, 4 mM glutamine, 1 mM pyruvate, 2% fatty acid free BSA, and the plate was degassed in a non-CO_2_ incubator at 37 °C for 1 h. After measuring basal oxygen consumption rate (OCR) in a Seahorse XF Pro Extracellular Flux Analyzer (Agilent Technologies, Santa Clara, CA, USA), cells were sequentially treated with oligomycin (5 µM), isoproterenol (0.5 µM), FCCP (7.5 µM) and antimycin A (5 µM) [12]. Sample size was determined based on previous experiments performed in our laboratory. Only wells with increased OCR after FCCP injection were analyzed. Outliers identified by ROUT analyses were excluded. Basal, proton leak-linked OCR and coupling efficiency were calculated according to manufactures’ equations.

### p53 depletion in adipocytes

For details please consult Supplementary Material and Methods.

### Data analysis

Data are presented as means ± SEM. Shapiro–Wilk test was used to assess normal distribution. When comparing two groups, Mann–Whitney test was used for not normally and unpaired two-tailed Student’s *t* test (with Welch’s correction in case of unequal variances) for normally distributed data. When comparing more than two groups, two-way ANOVA with Tukey’s multiple comparison test was used. In human studies, linear relationships were assessed by Spearman correlation. Statistical tests were calculated using GraphPad Prism (GraphPad Software, San Diego, CA, USA).

## Results

### Fas regulates p53 protein levels in adipocytes

**Fig. 1:**
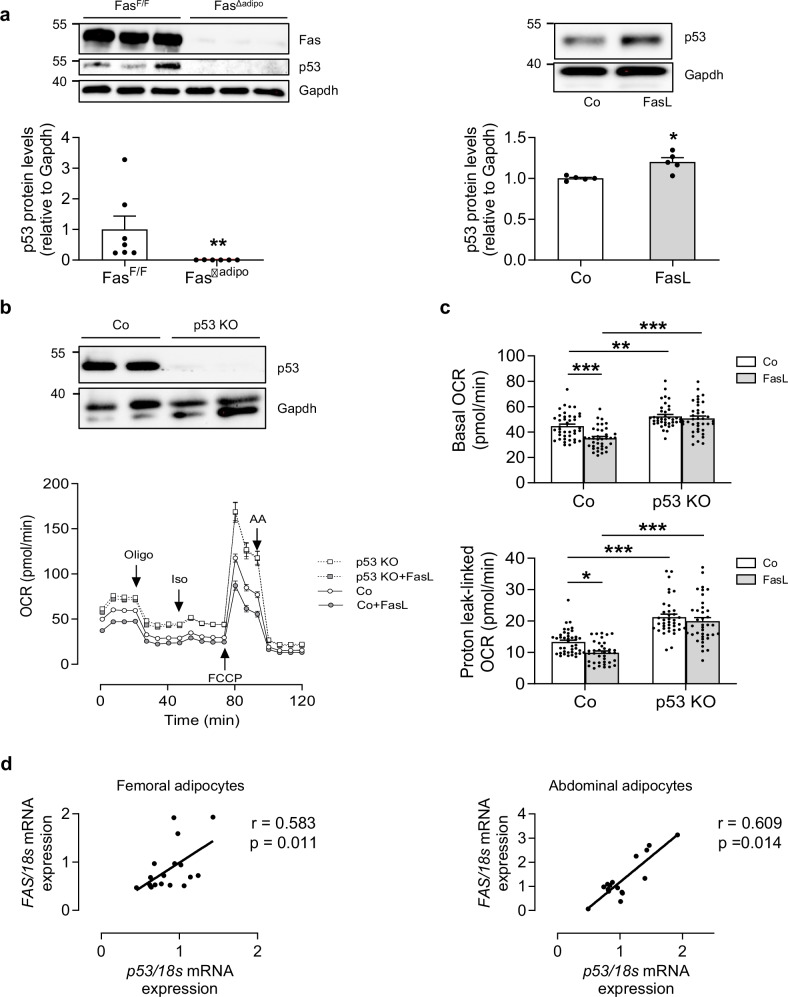
Fas regulates p53 protein levels in adipocytes. **a** Left panel: Western blot and quantification of Fas and p53 protein levels in white adipocytes harvested from HFD-fed Fas^F/F^ and Fas^Δadipo^ mice. *n* = 6–7 mice per group. **p < 0.01. Right panel: Western blot and quantification of p53 protein levels in subcutaneous white adipocytes treated with or without 0.4 ng/ml FasL for 24 h. *n* = 5 cell culture wells of 3 independent experiments. *p < 0.05. **b** Representative Western blot and oxygen consumption rate (OCR; *n* = 37–41 cell culture wells of 2 independent experiments) in control (Co) and p53-depleted (p53 KO) subcutaneous adipocytes treated with or without 0.4 ng/ml FasL for 72 h. Co vs. Co + FasL, *p* < 0.001; p53 KO vs. p53 KO + FasL, *p* = 0.43; Co vs. p53 KO, *p* < 0.001. **c** Basal and proton leak-linked respiration calculated from OCR data. *p < 0.05, **p < 0.01, ***p < 0.001. **d** Scatter plot and correlation coefficient (*r*) of *FAS* and *p53* mRNA expression in differentiated human mesenchymal stem cells derived from paired femoral (*n* = 18) and abdominal (*n* = 16) subcutaneous adipose tissue samples. Statistical tests used: Mann–Whitney (left panel) and Student’s *t* test (right panel) for (**a**); two-way ANOVA for (**b**, **c**); Spearman correlation for (**d**).

We first analyzed p53 protein levels in primary adipocytes isolated from adipocyte-specific Fas knockout mice. As depicted in Fig. 1a, p53 protein abundance was markedly reduced in HFD-fed knockout compared to control mice. Conversely, treatment of subcutaneous white adipocytes [13] with non-apoptotic concentrations of Fas ligand (FasL) [1] significantly increased p53 protein levels (Fig. 1a). These data indicate that Fas regulates p53 levels. Next, we aimed to investigate whether the Fas-p53 axis affects energy expenditure in adipocytes. To this end, Seahorse experiments were performed in FasL-treated adipocytes with or without CRISPR-Cas9-mediated knockout of p53 (Fig. 1b). Confirming previous findings [7], p53-knockout significantly increased OCR (Fig. 1b). The latter was paralleled by lower coupling efficiency and elevated protein levels of uncoupling protein 1 (UCP1) (Supplementary Fig. 1a, b). Importantly, FasL treatment significantly reduced basal and proton leak-linked OCR in control but not in p53-knockout cells (Fig. 1c), indicating that activation of Fas reduces energy expenditure in a p53-dependent manner.

To further investigate whether Fas regulates p53 levels in human adipocytes, we determined gene expression of *FAS* and *p53* in differentiated human multipotent femoral and abdominal adipose-derived stem cells (hMADS). As depicted in Fig. 1d, we found a significant positive correlation between *FAS* and *p53* mRNA expression in hMADS from both fat depots, suggesting that Fas may be a positive regulator of p53 in human adipocytes.

### p53 in human WAT correlates positively with FAS and BMI

**Fig. 2:**
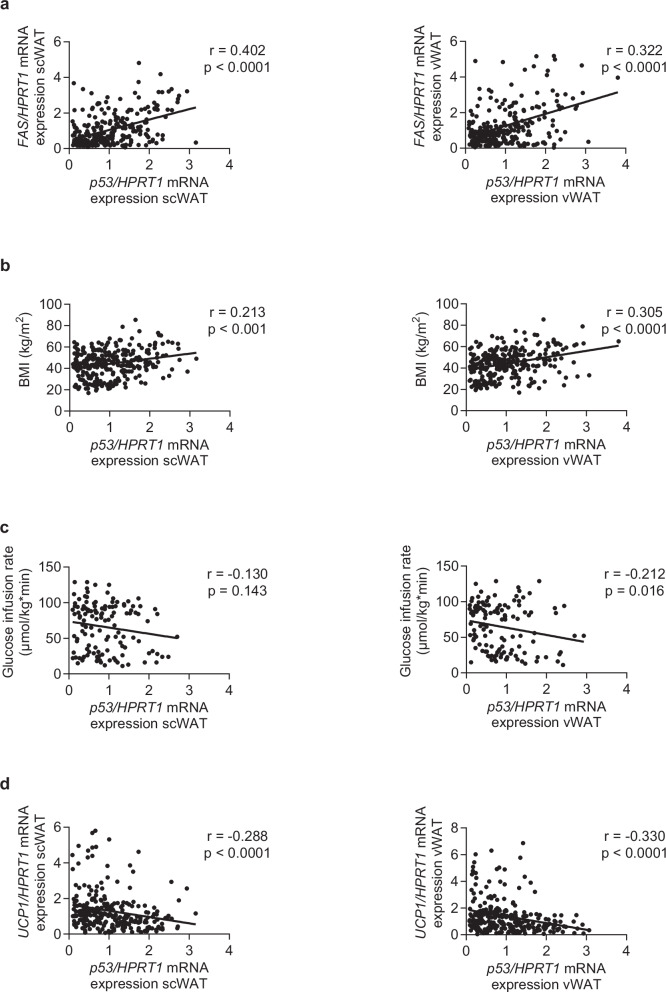
*p53* expression in human WAT correlates positively with *FAS* and BMI. **a** Scatter plot and correlation coefficient (*r*) of subcutaneous (sc; *n* = 254) or visceral (v; *n* = 250) WAT *FAS* mRNA and *p53* mRNA expression. **b** Scatter plot and correlation coefficient (*r*) of *p53* expression in scWAT (*n* = 281) or vWAT (*n* = 284) and BMI. **c** Scatter plot and correlation coefficient (*r*) of *p53* expression in scWAT (*n* = 129) or vWAT (*n* = 128) and glucose infusion rate during hyperinsulinemic-euglycemic clamps. **d** Scatter plot and correlation coefficient (*r*) of scWAT (*n* = 255) or vWAT (*n* = 265) *UCP1* and *p53* mRNA expression. Statistical tests used: Spearman correlation.

Next, we aimed to investigate whether *p53* expression in human WAT correlates with measures of obesity and insulin resistance. Of note, the positive correlation between *FAS* and *p53* expression in hMADS (Fig. 1d) was confirmed in human abdominal subcutaneous as well as in visceral WAT (Fig. 2a). Importantly, *p53* and *FAS* mRNA expression in both WAT depots correlated positively with BMI (Fig. 2b and Supplementary Fig. 2a). Moreover, we found a significant negative correlation between glucose infusion rates (GIR) during hyperinsulinemic-euglycemic clamps and *p53* as well as *FAS* expression in visceral WAT, while such correlation was significant for *FAS* but not *p53* in subcutaneous WAT (Fig. 2c and Supplementary Fig. 2b). In addition, *p53* expression in both WAT depots correlated significantly negative with *UCP1* (Fig. 2d).

## Discussion

The presented findings in mice and humans suggest that Fas regulates p53 levels in adipocytes, and may thereby negatively impact body weight gain and glucose metabolism. The regulatory role of Fas is supported by the findings that Fas activation increased p53 protein levels in adipocytes and that Fas-depleted adipocytes revealed markedly reduced p53 levels. In line, *FAS* correlated positively with *p53* expression in differentiated adipose-derived mesenchymal stem cells as well as human WAT of different anatomical locations. Thus, elevated Fas expression in WAT of mice and men with obesity may underlie the parallel increase in p53 levels [2–6]. Our data reveal significant correlations between BMI and *p53* expression in human subcutaneous as well as visceral WAT and are in contrast with previous findings reporting a positive correlation between BMI and *p53* expression in omental but not subcutaneous WAT [5]. Further studies are needed to better understand these discrepant findings as well as the clinical significance of the present results.

Seahorse analysis revealed that p53 depletion increased mitochondrial oxygen consumption in cultured adipocytes, whereas Fas activation reduced the latter in a p53-dependent manner. Hence, reduced p53 concentrations in adipocytes may be at least partly responsible for the higher energy expenditure and, hence, blunted body weight gain in HFD-fed adipocyte-specific Fas knockout mice [1]. Since Fas activation reduced uncoupling protein 1 (UCP1) content in adipocytes [1], further studies may unravel whether the inhibitory effect of the Fas-p53 pathway on oxygen consumption is mediated via a reduction in UCP1 concentration and/or other molecular pathways. In support of the former, we report herein that *p53* expression correlates positively with *FAS* but negatively with *UCP1* in human WAT.

Possibly, the stronger correlation between peripheral insulin sensitivity and *p53* expression in visceral compared to subcutaneous WAT may be explained by the fact that visceral fat is more prone to obesity-induced inflammation. The latter is an important driver of obesity-induced insulin resistance [14, 15] and may be further enhanced by p53. Indeed, expression of *p53* in omental WAT was positively associated with WAT inflammation (i.e., expression of TNFα and the macrophage marker CD68) but negatively with HbA1c [5]. Along the same line, reduced p53 levels in HFD-fed adipocyte-specific Fas knockout mice may have contributed to reduced WAT inflammation and, consequently, improved insulin sensitivity in these animals [1].

In conclusion, the present study identifies Fas as a regulator of p53 levels in adipocytes. Consequently, the detrimental effects of Fas on glucose homeostasis and energy metabolism may at least partly be mediated by p53.

## Supplementary information

Supplemental Material

Supplemental Figures 1-2

## Data Availability

The data supporting the findings of this study are available within the article or from the corresponding author upon reasonable request.
