# Supplementary material for: Expression of p53 in human adipose tissue correlates positively with FAS and BMI
Source: Int J Obes (Lond). 2024 Dec 1;49(4):737–41. doi: 10.1038/s41366-024-01691-4 (PMC11999857; doi:10.1038/s41366-024-01691-4)
Supplement: Supplementary file 1 — Supplemental Material [file 41366_2024_1691_MOESM1_ESM.docx]

**Material and Methods**

*Adipose tissue needle biopsies*

AT specimens were collected 6 to 8 cm lateral from the umbilicus and from the anterior site of the upper leg, respectively, under local anaesthesia (1% lidocaine) after an overnight fast. Biopsy specimens were immediately rinsed with sterile saline and visible blood vessels were removed with sterile tweezers. Next, human multipotent adipose-derived stem cells (hMADS), an established human white adipocyte model^1^, were isolated as described before^2, 3^. Cells were seeded at a density of 2000 cells/cm^2^ and kept in proliferation medium for seven days and thereafter in differentiation medium for 14 days. Total RNA was extracted from hMADS cells using TRIzol reagent (Invitrogen, Breda, Netherlands), and SYBR-Green-based real-time PCRs were performed using an iCycler (Bio-Rad, Veenendaal, Netherlands) to assess *Fas* and *p53* mRNA expression (same primers as used for subcutaneous WAT; see below). Results were normalized to 18S ribosomal RNA as a housekeeping gene (fw: AGTTAGCATGCCAGAGTCTCG; rv: TGCATGGCCGTTCTTAGTTG).

*Western Blotting*

Differentiated subcutaneous adipocytes^4^ were treated with 0.4 ng/ml FasL (Enzo Life Sciences AG, Lausen, Switzerland) or vehicle for 24 hours. Adipocytes were lysed and Western blotting was performed as described^5^. The following primary antibodies (diluted 1:1000) were used: Fas, 05-351 (Millipore, Darmstadt, Germany); p53, #2524 (Cell Signalling, Danvers, MA, USA), UCP1, PA1-24894 (Thermofisher Scientific, Waltham, MA, USA) and GAPDH, 10494-1-AP (Proteintech, Manchester, UK).

*p53 depletion in adipocytes*

An oligonucleotide for the cloning of a sgRNA targeting murine *Trp53* (sequence: 5'-T CCC CAG CAT CTT ATC CGG G-3') was synthesized (Microsynth AG, Switzerland), annealed and cloned into pL-CRISPR.SFFV.GFP (Addgene plasmid #57827). Plasmids were propagated in One ShotTM Stbl3TM E. coli bacterial strains (ThermoFisher Scientific). For generation of *Trp53^KO^* pre-adipocytes, replication-defective, third-generation lentiviral vectors were used. In brief, HEK293T cells were transfected with the transfer plasmid containing the DNA sequence of interest as well as with psPAX2 packaging and pCAG-VSV-G envelope plasmids (both kindly provided by Dr. Patrick Salmon, University of Geneva, Switzerland). JetPRIME® transfection reagent (Polyplus transfection, Illkirch, France) was used to enhance transfection efficacy. Viral particles were harvested 2 days later and concentrated with Peg-itTM (System Biosciences, Palo Alto, CA, USA). Concentrated viral particles were stored at -80°C until further use. Pre-adipocytes were transduced with lentiviral supernatants of either the vector containing the sgRNA sequence (p53 KO) or with the empty vector carrying Cas9 only and GFP (Co). Fluorochrome-expressing cells were sorted using a FACSAria (Becton Dickinson, Franklin Lakes, NJ, USA). Pre-adipocytes were not authenticated and not tested for mycoplasma. Validation of successful gene editing was confirmed by immunoblotting.

**References**

1. Jocken JW, Goossens GH, Popeijus H, Essers Y, Hoebers N, Blaak EE. Contribution of lipase deficiency to mitochondrial dysfunction and insulin resistance in hMADS adipocytes. *Int J Obes (Lond)* 2016; **40**(3)**:** 507-13.

2. Vogel MAA, Jocken JWE, Sell H, Hoebers N, Essers Y, Rouschop KMA *et al.* Differences in Upper and Lower Body Adipose Tissue Oxygen Tension Contribute to the Adipose Tissue Phenotype in Humans. *J Clin Endocrinol Metab* 2018; **103**(10)**:** 3688-3697.

3. Lempesis IG, Hoebers N, Essers Y, Jocken JWE, Dubois LJ, Blaak EE *et al.* Impaired mitochondrial respiration in upper compared to lower body differentiated human adipocytes and adipose tissue. *J Clin Endocrinol Metab* 2024; online ahead of print.

4. Wueest S, Scaffidi C, van Krieken PP, Konrad NK, Koch C, Wiedemann MSF *et al.* Fas (CD95) expression in adipocytes contributes to diet-induced obesity. *Obesity (Silver Spring)* 2024; **32**(10)**:** 1812-1818.

5. Scaffidi C, Srdic A, Konrad D, Wueest S. IL-27 increases energy storage in white adipocytes by enhancing glucose uptake and fatty acid esterification. *Adipocyte* 2023; **12**(1)**:** 2276346.
