# Supplementary material for: Expression of p53 in human adipose tissue correlates positively with FAS and BMI
Source: Int J Obes (Lond). 2024 Dec 1;49(4):737–41. doi: 10.1038/s41366-024-01691-4 (PMC11999857; doi:10.1038/s41366-024-01691-4)
Supplement: Supplementary file 2 — Supplemental Figures 1-2 [file 41366_2024_1691_MOESM2_ESM.pdf]

Expression of p53 in human adipose tissue correlates positively with FAS and BMI

Stephan Wueest, Chiara Scaffidi, Pim P. van Krieken, Nils K. Konrad, Christian Koch, Ioannis G. Iempesis, Jonas Fullin, Konstantinos N. Manolopoulos, Steffen Böttcher, Gijs H. Goossens, Matthias Blüher, Daniel Konrad

Supplementary Figures 1-2

Supplementary Figure 1

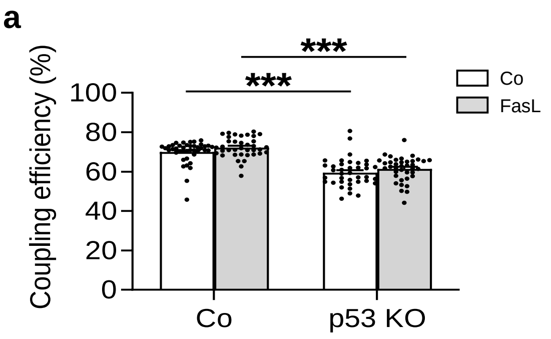

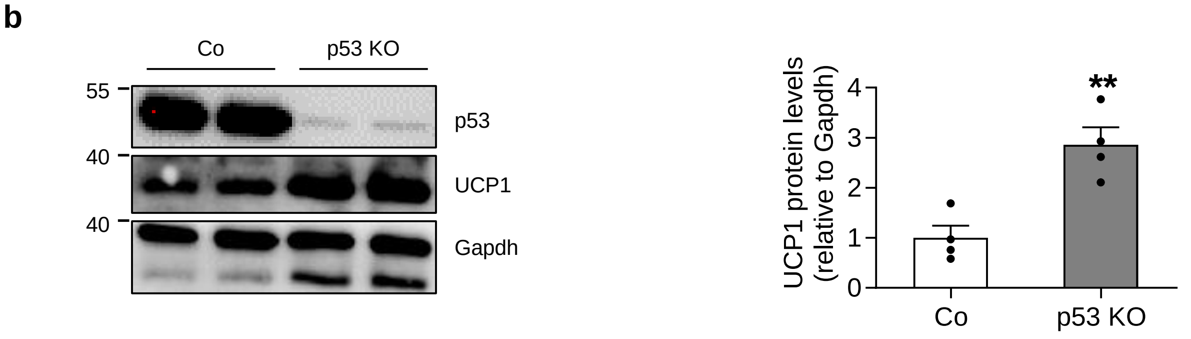

Decreased coupling efficiency in p53 KO cells

(**a**) Coupling efficiency calculated from OCR data. ***p<0.001. (**b**) Representative Western blot (left panel) and UCP1 quantification (right panel; n=4 cell culture wells of 2 independent experiments) in control (Co) and p53-depleted (p53 KO) subcutaneous adipocytes treated with isoproterenol for 6 hours. Statistical tests used: two-may ANOVA for **a**; Student's *t* test for **b**.

Supplementary Figure 2

a

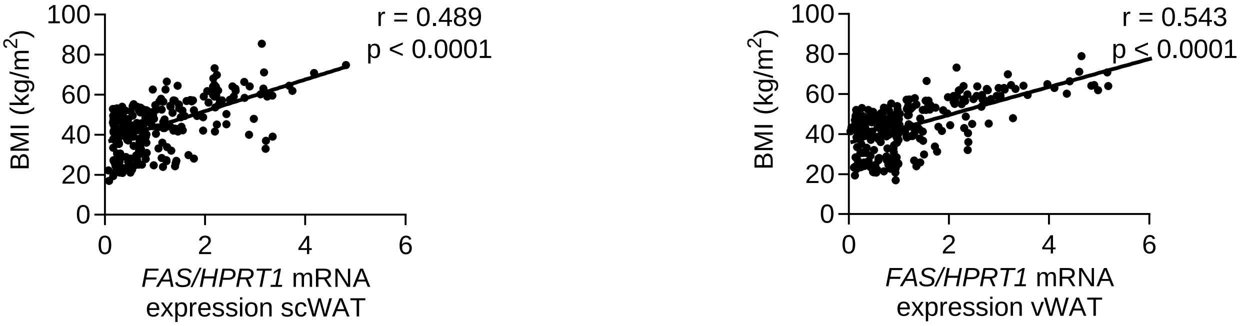

b

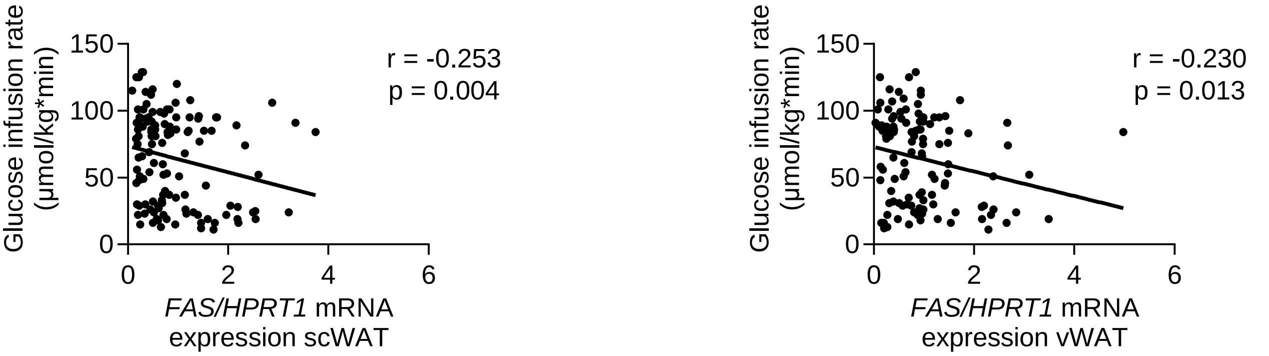

***FAS* expression in human WAT correlates positively with BMI and negatively with glucose infusion rate**
(**a**) Scatter plot and correlation coefficient (r) of subcutaneous (sc; n=270) or visceral (v; n=266) WAT *FAS* mRNA and BMI. (**b**) Scatter plot and correlation coefficient (r) of *FAS* expression in scWAT (n=126) or vWAT (n=115) and glucose infusion rate during hyperinsulinemic-euglycemic clamps.
